# New soft tissue data of pterosaur tail vane reveals sophisticated, dynamic tensioning usage and expands its evolutionary origins

**DOI:** 10.1101/2024.07.01.601487

**Published:** 2024-07-03

**Authors:** Natalia Jagielska, Thomas G. Kaye, Michael B. Habib, Tatsuya Hirasawa, Michael Pittman

**Affiliations:** 1 School of GeoSciences, The University of Edinburgh, Edinburgh, United Kingdom.; 2 Foundation for Scientific Advancement, Sierra Vista, Arizona, United States.; 3 Department of Medicine, University of California Los Angeles, Los Angeles, United States; 4 Department of Earth and Planetary Science, Graduate School of Science, The University of Tokyo, Tokyo, Japan; 5 School of Life Sciences, The Chinese University of Hong Kong, Shatin, Hong Kong SAR, China.

## Abstract

Pterosaurs were the first vertebrates to achieve powered flight. Early pterosaurs had long stiff tails with a mobile base that could shift their center of mass, potentially benefiting flight control. These tails ended in a tall, thin soft tissue vane that would compromise aerodynamic control and efficiency if it fluttered during flight like a flag in the wind. Maintaining stiffness in the vane would have been crucial in early pterosaur flight, but how this was achieved has been unclear, especially since vanes were lost in later pterosaurs and are absent in birds and bats. Here we use Laser-Stimulated Fluorescence imaging to reveal a cross-linking lattice within the tail vanes of early pterosaurs. The lattice supported a sophisticated dynamic tensioning system used to maintain vane stiffness, allowing the whole tail to augment flight control and the vane to function as a display structure.

## Introduction

**Figure 1.**
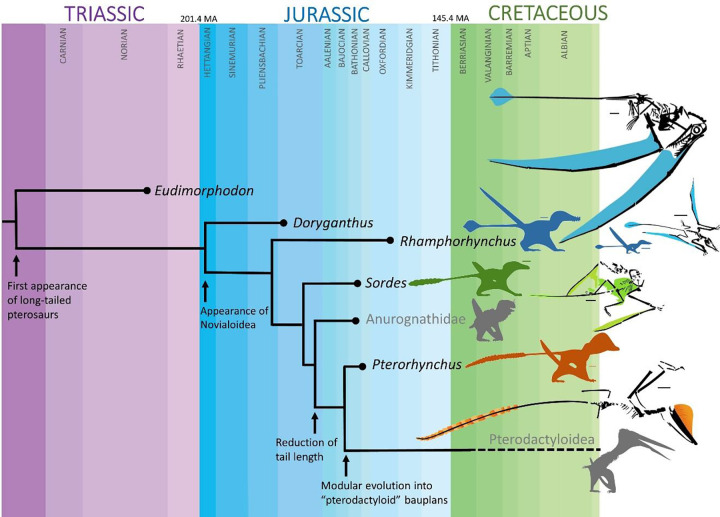
Long-tailed early-diverging non-pterodactyloid pterosaurs had diverse tail vanes but these disappeared in later-diverging short-tailed pterodactyloids. Blue, ontogenetic morphs of *Rhamphorhynchus muensteri*: NMS G.1994.13.1 and BSP 1938 I 503a. Green, *Sordes pilosus* PIN 2585/3. Orange, *Pterorhynchus wellnhoferi* CAGS02-IG-guasa-2/DM608. Scale bars are 3 cm.

Pterosaurs were the first vertebrates to achieve powered flight (Palmer 2017). The first pterosaurs, the non-pterodactyloids, had long, stiff tails with a mobile base (Frey, Tischlinger et al. 2003), similar to some dinosaurs like *Velociraptor* (Persons IV and Currie 2012). Many of these tails end in a soft tissue ‘vane’ (Marsh 1882, Döderlein 1929, Frey, Tischlinger et al. 2003) (Fig. 1), which may have contributed to passive stability in flight.

A primary role in display has also been suggested (O’Brien, Allen et al. 2018), given ontogenetic shape changes in the vane and the fact that, unlike most aircraft, flying animals do not need vertical control surfaces to be yaw-stable during turns (Bowers, Murillo et al. 2016).

The vanes have been interpreted as steering aids (Frey, Tischlinger et al. 2003). The length and stiffening of the tails suggest that they might have been important in early pterosaurs for control based on mass shifting or inertial control, as purported for terrestrial theropods with convergent tails (Persons IV and Currie 2012). Such dynamic control could greatly improve maneuverability and/or stability. However, vane fluttering would be extremely costly and destabilising unless the vane was tensioned while under aerodynamic load. Tail vanes feature thick, evenly spaced, internal structures roughly perpendicular to the caudal series (Döderlein 1929), that are said to resemble neural spines and haemal arches (Marsh 1882). These structures are presumed to have minimised fluttering and prevented buckling in the same way that spars, ribs, stringers, and longerons do in airplane wings and tail-fins, but others have proposed that they were flexible and cartilaginous (Marsh 1882), especially since their preserved appearance varies. Here we use Laser-Simulated Fluorescence (LSF) imaging of *Rhamphorhynchus* specimens from the Upper Jurassic Solnhofen Limestones (Kaye, Falk et al. 2015, Pittman, Barlow et al. 2021) to investigate the vane’s structural properties, explore its usage, its evolutionary origins and the context for its disappearance in later pterodactyloids (Frey, Tischlinger et al. 2003).

## Results

**Figure 2.**
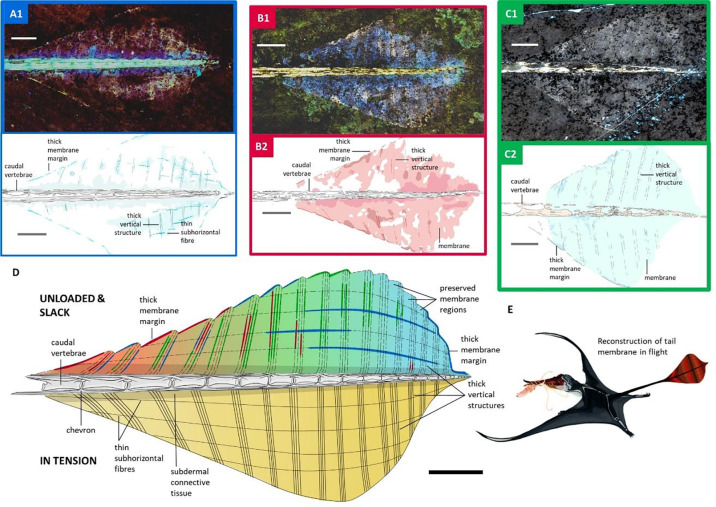
Tail vane of *Rhamphorhynchus muensteri*. **A1.** LSF image of NHMUK PV OR 37787. **A2.** Line drawing of LSF image of NHMUK PV OR 37787. **B1.** LSF image of NHMUK PV OR 37003. **B2.** Line drawing of LSF image of NHMUK PV OR 37003. **C1.** LSF image of NMS G.1994.13.1. **C2.** Line drawing of LSF image of NMS G.1994.13.1. **D.** Interpretative line drawing of *Rhamphorhynchus muensteri* tail vane unloaded and slack as well as in tension. Combines LSF results of NHMUK PV OR 37003 and 37787 as well as NMS G.1994.13.1 (A1-C2). **E.** Life reconstruction of *Rhamphorhynchus muensteri* using its tail vane during flight. All scale bars are 1 cm.

Over 100 Solnhofen pterosaur fossils were examined for well-preserved tail vanes using an ultraviolet torch. Four exceptional specimens were then imaged under Laser-Stimulated Fluorescence (LSF). Three specimens exhibited tail vanes under white light but the vane of NHMUK PV OR 37003 was only visible under LSF. LSF confirmed the soft tissue extent of the vanes and revealed hidden anatomical details, especially in NHMUK PV OR 37003 and 37787 and NMS G.1994.13.1 (Fig. 2A–C), where vane areas fluoresced pink and white, indicating soft tissue preservation (Pittman, Barlow et al. 2021). Tail vanes are sub-symmetrical and diamond-shaped in NHMUK PV OR 37003 and 37787 and NMS G.1994.13.1 with a length of 700 mm, 750 mm and 720mm making up 21%, 22% and 21% of the total tail length of 320 mm, 362 mm and 348 mm respectively (Fig. 2). At its widest point, about two-thirds along its length, the vane is 41 mm across in NHMUK PV OR 37003 and 37787 but the widest in NMS G.1994.13.1 (55 mm), even wider than in BSP 1907 I 37 (46 mm) and almost twice as wide as YPM 1778 (30 mm). Under LSF, partial edges of the vane are visible, along with at least 17 relatively straight structures in NHMUK PV OR 37787 (10+ in NHMUK PV OR 37003 and 11+ in NMS G.1994.13.1); projecting vertically, near-perpendicularly to the tail skeleton (based on position of chevron bones e.g., NHM PV OR 37787). In NHMUK PV OR 37003 and 37787 and NMS G.1994.13.1, these are relatively thick (0.6 – 1 mm) and appear to be hollow, suggesting they were rod-like, and were arranged in parallel ~3 – 8 mm apart. In NHMUK PV OR 37003 and 37787 they are rarely preserved dead-straight, but are straighter in NHM G.1994.13.1, especially anteriorly. In YPM 1778 and BSP 1907 I 37 the vertical structures show more pronounced undulations giving them a sigmoidal morphology. To our knowledge, in NHMUK PV OR 37003 alone, there is a second layer of thinner and more numerous fibres that run across the thick vertical structures, subparallel to the long axis of the tail and become more and more closely spaced as they reach the tail tip. Together, the vertical structures and subhorizontal fibres form a cross-linked lattice. The thick outer margin of the vane is undulated in dorsal view in both NHMUK PV OR 37003 and 37787, with a trough ~0.7 mm deep where the thick vertical structures meet the margin and a convex peak roughly mid-way between each pair of vertical structures. A similarly thick outer margin is also observed anteriorly on the tail vane of NMS G.1994.13.1 and BSP 1907 I 37.

## Discussion

LSF imaging demonstrates that the “problem” of vane flutter in early pterosaurs was solved using two sets of tensile-loading structures. One set are thicker, hollow, regularly-spaced, tube-like structures with a vertical long axis (Fig. 2). The second set are thinner more numerous subhorizontal fibres that criss-cross the thick vertical structures and transfer tension into the tail tip (Fig. 2). The cross-linked lattice worked to prevent flutter by limiting the degree to which the tail vane could bend out of plane. Mediolateral deviations would bend the thicker vertical structures and therefore be resisted by the tensile strength of those fibres. Stretching of the vane would spread the vertical structures, and this would load the cross-linking fibres running to the tail tip - greatly limiting the degree to which the structures could bend apart and therefore limiting stretch of the vane (providing dynamic stiffness).

While it may be tempting to think of the thick vertical structures as compression-loading sail battens, this does not seem to be a major loading regime. If the vertical structures loaded in axial compression to maintain vane shape, we would expect the peaks of the undulating vane edge contour to be aligned with the fibre tips: in the way that battens align with the convexity on a racing sail trailing edge (Calì, Sapienza et al. 2022). Notably, it is the concavities that align with each structure tip, indicating that, under load, the spaces between the structures stretched until the outer edge was linear (Fig. 2D), with the fossilised position preserving the unloaded slack condition (Fig. 2D).

Our results suggest that the tail vane maintained effective stiffness dynamically with internal tension of a cross-linked lattice that minimised excessive vane flutter and associated drag production. This structural integrity would have permitted the vane to be to be recruited in stabilisation, likely working in collaboration with uropatagium/cruropatagium and webbed feet when present, and as an effective display structure (Fig. 2E). This tensioning would also have allowed the tail, as a whole, to be used for mass-shifting-based aerodynamic control without incurring adverse effects of a fluttering vane during rapid tail motions. Tail vane shape changes through ontogeny (Bennett 1995, O’Brien, Allen et al. 2018) and between species (Marsh 1882, Döderlein 1929, Frey, Tischlinger et al. 2003), which underscores the importance of the tail vane in early pterosaur evolution. As pterodactyloid pterosaurs evolved a shorter body plan with an anterior center of mass and large heads with cranial crests as the primary display structures (Jagielska and Brusatte 2021), both the control and display functions of the tail were absorbed by the wings and head.

The new soft tissue information also provides clues about the evolutionary origins of the tail vane itself. The cross-linked lattice recognised in this study suggests that the tail vane of early pterosaurs developed from a single contiguous structure rather than a combined structure of scales or feather-like integuments. While the undulating vane edge (Fig. 2D) might reflect an epidermal patterning, the internal part of the tail vane was likely filled with connective tissue underneath the epidermal layer. The medial part of the tail vane, namely the periphery of the caudal vertebrae, has a different tone under fluorescence and the vertical structures lose clarity when compared to the lateral part of the tail vane (Fig. 2A, B). This potentially indicates a thicker subdermal connective tissue surrounding the caudal vertebrae. Therefore, the tail vane of pterosaurs consisted of bilateral fleshy folds on the end of the tail, comparable to the cetacean fluke that envelopes dense connective tissue (Gavazzi, Nair et al. 2024). The growth series of the tail vane shape in *Rhamphorhynchus muensteri* begins with an extended teardrop/oval shape, becoming diamond-shaped (Fig. 2A, B), and eventually triangular (Bennett 1995). These three shapes parallel the shape changes of the cetacean fluke during embryonic development (Štěrba, Klima et al. 2000). It is possible that both the pterosaur tail vane and the cetacean fluke evolved through a shared developmental mechanism, perhaps a co-option of the signaling pathway that drives appendage outgrowth (Gavazzi, Nair et al. 2024), eventually bringing about improved fluid dynamics of the limbs.

## Materials and Methods

Over 100 Solnhofen pterosaur fossils were examined for well-preserved tail vanes using an ultraviolet torch at the Bayerische Staatssamlung für Paläontologie (Munich), Museum für Naturkunde (Berlin), Jura Museum (Eichstätt), Natural History Museum (London), National Museum of Scotland (Edinburgh) and Royal Ontario Museum (Toronto). Four exceptional specimens were imaged under Laser-Stimulated Fluorescence (LSF) (Kaye, Falk et al. 2015) at the Natural History Museum, National Museum of Scotland, and the Royal Ontario Museum. LSF involved projecting a 405 nm violet laser diode from a line lens and scanning it over the specimens in a dark room following standard laser safety protocol. Long exposure photographs over 30 seconds were taken with a Nikon digital single-lens reflex camera fitted with a 425 nm laser blocking filter. LSF images were then postprocessed for equalisation, saturation, and colour balance across the entire images in Adobe Photoshop CS6. For more details see Extended Methods in Supplementary File 1.

## Data Availability

All relevant data is provided in the manuscript and Supplementary File 1.
